# Wet-Dry Cycling Delays the Gelation of Hyperbranched Polyesters: Implications to the Origin of Life

**DOI:** 10.3390/life9030056

**Published:** 2019-07-01

**Authors:** Irena Mamajanov

**Affiliations:** Earth-Life Science Institute, Tokyo Institute of Technology, Meguro, Tokyo 152-8550, Japan; irena.mamajanov@elsi.jp; Tel.: +81-3-5734-3414

**Keywords:** hyperbranched polyester, functional polymer, chemical evolution, wet-dry cycle, gelation prevention, condensation polymer, origin of life

## Abstract

In extant biology, biopolymers perform multiple crucial functions. The biopolymers are synthesized by enzyme-controlled biosystems that would not have been available at the earliest stages of chemical evolution and consist of correctly sequenced and/or linked monomers. Some of the abiotic “messy” polymers approximate some functions of biopolymers. Condensation polymers are an attractive search target for abiotic functional polymers since principal polymers of life are produced by condensation and since condensation allows for the accurate construction of high polymers. Herein the formation of hyperbranched polyesters that have been previously used in the construction of enzyme-like catalytic complexes is explored. The experimental setup compares between the branched polyesters prepared under mild continuous heating and the wet-dry cycling associated with environmental conditions, such as dew formation or tidal activities. The results reveal that periodic wetting during which partial hydrolysis of the polyester occurs, helps to control the chain growth and delays the gel transition, a mechanism contributing to the tar formation. Moreover, the NMR and mass spec analyses indicate that continuously dried samples contain higher quantities of crosslinked and macrocyclic products, whereas cycled systems are enriched in branched structures. Ostensibly, environmental conditions have the ability to exert a rudimentary pressure to selectively enrich the polyesterification products in polymers of different structures and properties. At the early stages of chemical evolution, in the absence of biological machinery, this example of environmental control could have been for selectivity in chemical systems. As expected in marginally controlled systems, the identification of each component of the heterogeneous system has proved challenging, but it is not crucial for drawing the conclusions.

## 1. Introduction

In modern biological systems, biopolymers perform a multitude of functions. The major biopolymers (i.e., polypeptides, nucleic acids, polysaccharides) are characterized by a specific monomer sequencing and folding to achieve the necessary functions. In biochemistry, the synthesis of biopolymers is accomplished through intricate enzyme-controlled processes [1], that would not have been available at the earliest stages of chemical evolution. The abiotic synthesis of biopolymers is an active area of research. It has long been recognized that abiotic reactions can produce many monomers of biopolymers. For example, the Miller−Urey experiment showed that some of the simpler amino acids (e.g., glycine and alanine) are readily formed in a model prebiotic atmosphere [2,3]. Besides, amino acids used in life today have been found in carbonaceous meteorites that are more than 4 billion years old [4,5,6]. Different biopolymer blocks, including sugars and nucleobases, are also formed in a variety of model prebiotic reactions [7,8,9,10] and found in some meteoritic samples [11]. Several high-yielding abiotic processes of coupling of amino acids [12,13] and nucleotides [14,15,16] using activating agents have been proposed. These models, however, still lack mechanisms for a robust sequence and structure control of the products limiting the plausibility of functional biopolymer formation.

As an alternative approach to the abiotic synthesis of functional polymers chemically different from those of contemporary biopolymers has been considered [17,18,19]. It has been long appreciated that model prebiotic systems yield large amounts of intractable tarry polymeric material [20,21]. Some polymeric components of the tarry material with structures different from those of existing biopolymers are possibly capable of approximating some of the biological functions. Examples of this approach include the catalytic function of proteinoid microsphere structures formed upon non-specific thermal condensation of certain amino acids [22,23,24].

Prebiotically plausible condensation polymers are a compelling system for the study of the chemical evolution of functional polymers. Firstly, principal polymers of extant life are synthesized via condensations reactions. Secondly, the step-growth mechanism of condensation allows for control over each step of polymerizations. This property allows for the construction of high polymer with accurately known structures [25]. Unlike the addition polymerization under which the chain elongation can occur only by the addition of a single monomer, condensation reaction can occur between existing polymer species combining properties of different polymeric domains into one product. Furthermore, condensation reactions are often reversible; a polymeric product could break down into oligomeric building blocks that in turn could recombine into a polymer with novel properties.

The bonds linking the monomers of many condensation biopolymers are characterized by the positive Gibbs free energy of formation in the aqueous environment of the cytoplasm. For example, the energy of the amide bond in a polypeptide backbone ranges from +2 to +4 kcal/mol in aqueous solution [26]. One exception is the ester bond found in naturally occurring polymers, i.e., cutin and polyhydroxybutyrate, is characterized by slightly negative bond energy (~−1 kcal/mol under physiological conditions [27]). As polyester synthesis is more thermodynamically favorable than that of a polypeptide, polyesters have been hypothesized to have preceded peptides by Orgel [28], and this notion is perhaps supported by the demonstrated ability of the ribosome to catalyze α-hydroxy acid coupling [29,30]. Glyceric acid has been previously shown to polymerize and form polyester chains of up to 25 residues under acidic conditions and moderate heating. Poly-glyceric acids prepared from optically pure and racemic glyceric acid have different solubilities suggesting the possibility of chiral selection [31]. We have shown that poly-malic acid can be formed under mild dry heating and maintained in water solution while undergoing wet-dry cycling [32].

Controlling the sequence of monomer units is not the only means to derive function from biopolymers. In polysaccharides, different stereochemistry of the glycosidic bonds, as well as linear or branched nature of the biomacromolecule, dictates its function. The polymers comprised solely of glucose monosaccharides are a compelling case in point. Cellulose, the major structural component of cell walls, is a linear polymer composed of repeated glucose units bonded by β-1,4 glycosidic bond. Cellulose is insoluble in water [33] and possesses high tensile strength [34]. In branched polysaccharides used as energy reserves, glycogen in animals, and amylopectin in plants, the glucose units are linked in α-1,4 glycosidic bonds with branching α-1,6 bonds. Both polymers are water soluble.

Highly or hyperbranched polymers (HBPs) have attracted significant attention from industrial, synthetic and biomedical communities due to their unique properties, such as low viscosity and solubility. HBPs are intrinsically globular and have a high propensity towards ligand binding either inside internal pockets with suitable environments or at the polymer surface that is characterized by a large number of potentially functional end groups [35]. Biomimetic catalytic properties of HBP and dendrimers, a regular subclass of HBP, are well documented [35,36,37,38]. We have previously demonstrated that straightforwardly synthesized mixture of amine-bearing hyperbranched polyesters (HBPEs) is capable of catalyzing the Kemp elimination process by modulating the polarity of the environments in the internal pockets of HBPEs [19]. Furthermore, HBPs, and HBPEs, in particular, can be prepared in the one-pot process [39,40,41] and under prebiotically plausible conditions [42].

**Scheme 1:**
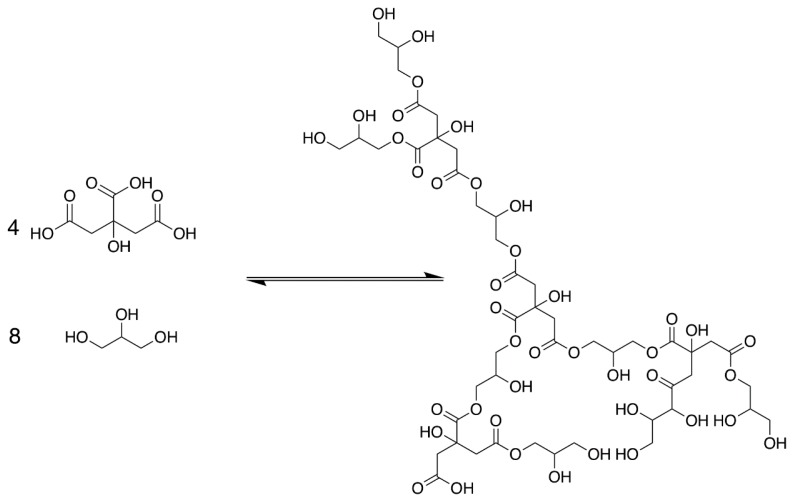
Schematic representation of citric acid and glycerol polyesterification. The formation of one of the possible oligomers comprised from four citric acid and eight glycerol monomers is depicted. The polymerization is of A_3_ + B_3_ type. Here A refers to carboxylic acid groups, B refers to alcohol groups, subscript refers to the number of the functional group in each monomer.

HBP synthesis commonly involves the coupling of monomers of AB_x_ type, where A and B refer to different mutually reactive functional groups. Flory’s statistical theory of mass distributions of three-dimensional polymers suggests that polymerization of monomers of AB_x_ type can proceed infinitely without the occurrence of gelation due to the formation of crosslinked networks [43]. Condensation of AB_x_ monomers has been used to prepare a wide range of polymeric products. For example, 3,5-bis(trimethylsylyloxy) benzoyl chloride and 3,5-dibromophenyl-boronic acid, AB_2_ type monimers, react to form hyperbranched polyphenylenes [44,45,46], whereas 2,2-bis(hydroxymethyl)propionic acid (AB_2_ type) is used to prepare hyperbranched polyesters [47,48]. Alternatively, HBPs can be synthesized starting from homofunctional monomers, some of which are more readily available commercially and are prebiotically relevant [49]. The present study focuses on a model polyesterification yielding branched polyesters synthesized from homofunctional monomers, glycerol and citric acid (Scheme 1).

HBP tend to undergo the process of gelation. Gelation occurs when a branched polymer forms an extensive network between strands through cross-linking [50].The gel transition is associated with a drastic change in polymer properties, such as viscosity and solubility [49].When the goal is to synthesize soluble catalysts, gelation is undesirable. Gelation is sharply dependent upon the degree of polymerization [50]. In synthetic chemistry, many methods exist for controlling and preventing gelation, such as a rigorous control over monomer stoichiometry [49,51] and the degree of polymerization [46,52], branching core inclusions [48], etc. The focus of this study is to explore a more straightforward prebiotically reasonable conditions of wet-dry cycling associated with, e.g., day-night, tidal or geyser activities as a method of HBPE chain growth control.

## 2. Materials and Methods

Reagent grade citric acid and glycerol were purchased from Sigma-Aldrich and used without further purification.

**Scheme 2:**
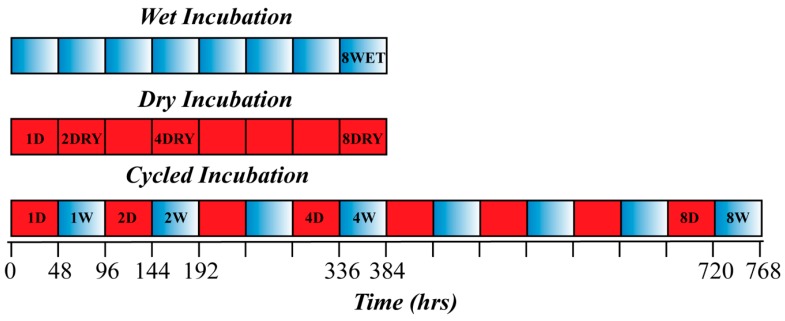
Schematic representation of experimental setup and the sampling schedule. Red squares represents the dry heating period, blue-shaded squares represent the wet incubation period. The samples were collected at the end of labeled periods.

A typical polyesterification reaction was conducted starting with 5ml of an aqueous solution containing 330 mM of citric acid and 660 mM glycerol. The pH of the solution was not adjusted; the pH was measured at 2 and remained unchanged throughout the polymerization process. The samples undergoing wet-dry cycle were allowed to air dry for 48 h at 85 °C, reconstituted, and incubated for 48 h at 75 °C. Sample tubes were removed at the end of first, second, fourth, and eighth periods of both wetting and drying. Continuously dried samples were allowed to air dry and incubated at 85 °C and sampled after 48, 96, 192, and 384 h. An additional sealed sample was incubated for 384 h. Schematic representation of the incubation and sampling schedule is depicted in Scheme 2. Prior to analysis, the dry samples were reconstituted and stirred at room temperature for 12 h. Aliquots were collected from all solutions, freeze dried, and dissolved in appropriate solvents.

For size exclusion chromatography (SEC) analysis, the reconstituted dry and wet samples were diluted 1:1 in deionized water. The analysis was performed on an Advanced Polymer Chromatography system (Waters Corporation, Manchester, UK) interfaced with a UV-vis detector equipped with Acquity APC AQ column [125 Å, 2.5 μm, 4.6 mm Å~ 30 mm, Waters Corp] and Acquity APC XT column (125 Å, 2.5 μm, 4.6 mm Å~ 30 mm, Waters Corp). An isocratic flow of 0.500 mL min^−1^ using H2O or 80%/20% THF/Methanol was utilized. The column temperature was set at 40 °C.

MALDI-MS spectra were collected on an ultrafleXtreme Bruker Daltonics MALDI-TOF-MS (Bruker Corporation, Billerica MA, USA) in positive ion mode. External mass calibration was conducted using standard peptide mixtures. Sample preparation matrix (trans-2-(3-(4-tert-butylphenyl)-2-methyl-2-propenylidene)malononitrile or (DCTB)) was dissolved 80%/20% THF/Methanol. Subsequently, the freeze-dried samples and the matrix were mixed at a 1:10 [*v*/*v*] ratio in advance and then the mixture was applied to the plate before analysis. ESI-MS spectra were collected on a Bruker micrOTOF II (Bruker Corporation, Billerica MA, USA) in positive ion mode. The samples were dissolved in 80%/20% THF/Methanol.

1H Nuclear Magnetic Resonance [NMR] spectra were recorded on a Bruker Avance 400 spectrometer (Bruker Corporation, Billerica MA, USA) at 25 °C. The spectra were collected employing 30° inversion pulses with 11s acquisition time, and 1s recycle delay.

## 3. Results

### 3.1. Polyesterification

Since this study intends to approximate prebiotically plausible conditions, the polymerization of citric acid and glycerol was conducted without an active water removal or catalyst addition. The starting solution for all polymerizations contained 2:1 ratio of glycerol to citric acid. Excess of glycerol has been previously shown to favor more soluble less cross-linked product [42,51]. Dry heating of the glycerol citric acid solution for the first 48 h (cycle 1D, Scheme 2) produced a clear gelatinous product. Similar clear glass like products formed after 96, 192, and 384 h of continuous drying (cycles 2DRY, 4DRY, 8DRY, Scheme 2). These products swelled in water but did not fully dissolve after stirring for 12 h. The sample 8DRY was subjected to wet incubation at 75 °C for 48 h, but failed to fully dissolve. For the wet-dry cycling experiment, the regime of 48 h of dry heating at 85 °C followed by 48 h of wet incubation at 75 °C for additional 48 h was selected. The citric acid-glycerol polymer has proven to be more resistant to hydrolysis than the previously investigated poly-malic acid system [32]. Whereas poly-malic acid polymer would fully hydrolyze after several hours of heating at 75 °C, substantial amounts of citric acid-glycerol polymeric material were still present after 48-h period of incubation. The hydrolysis of branched polymers is often retarded compared to linear polymers as dendritic spatial structures of the former limit the accessibility of water [53,54,55,56]. The systems that were subjected to periods of wet-dry cycled products remained fully soluble by the end of 384 h. The last dried sample (8D) was slow to dissolve; the solid did not fully dissolve following the typical sample workup, stirring for 12 hrs. The solid fully dissolved during the 48-h wet incubation at 75°C (equivalent to 8W sample). The observations suggest that continuously dried polymerization systems have undergone gelation much faster than the cycled ones. Little to no polymer was formed in the sample that have undergone continuous wet incubation suggesting that condensation only takes place during the drying periods.

### 3.2. Size-Exclusion Chromatography (SEC)

**Figure 1:**
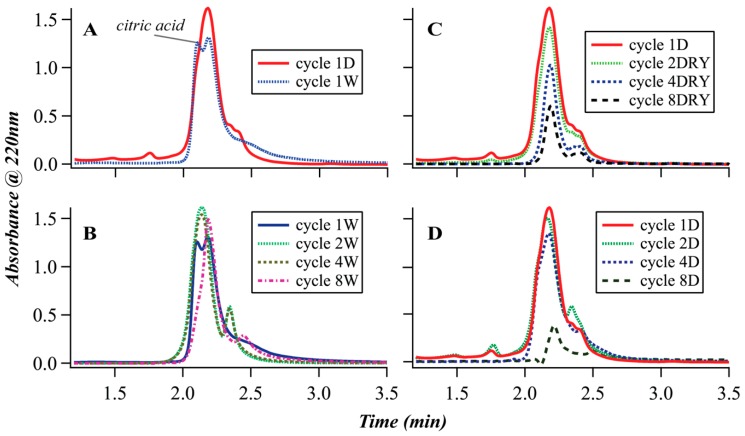
Size exclusion chromatography (SEC) analysis of the citric acid glycerol polymerization products (**A**) Comparison between the samples collected at the end of the first dry and wet cycle; (**B**) Comparison between the samples collected at the end of wet cycles (W series); (**C**) Comparison between samples of the DRY series; (**D**) Comparison of the samples collected at the end of dry period in the cycled series (D series).

SEC is a chromatographic technique yielding separation based on the hydrodynamic volume of analytes. The separation is triggered by the disparity in the interactions of the eluting particles within pores of the stationary phase unlike the surface interactions in other chromatographic methods. In principle, high molecular weight molecules, with large hydrodynamic volumes elute quickly, whereas smaller molecules experience more interactions with the stationary phase and elute later. In the case of linear polymers, the hydrodynamic volume is directly correlated to the molecular weight; thus, the molar masses of unknown linear polymers may be estimated relative to standards. In the case of branched and dendritic polymers, such correlation is challenging to draw [47]. Moreover, the nature of the terminating groups of branched polymers is expected to have a substantial effect on the polymer interaction with the solvent that, in turn, determines the hydrodynamic volume. The hydrodynamic volume of carboxylic acid terminated dendrimers in aqueous solution, for example, is strongly pH-dependent, and can change up to 50 % by merely changing the pH of the solution [57]. The SEC separation, in this case, is therefore based mainly on a complex relationship between the oligomer chemistry and architecture rather than the molar mass. The chromatographs shown in Figure 1 were analyzed using aqueous mobile phase. Better separation of the low molecular weight products was achieved using normal phase THF-methanol mobile phase [Figure S1], however the products only marginally dissolved in THF-methanol and caused on-column precipitation. Only small number of samples was analyzed using normal phase SEC.

The chromatographs in Figure 1 represent the polymeric samples produced during different experimental cycles (Scheme 2). Panel 1A depicts the chromatographs of the samples that have undergone one cycle of drying (1D or DRY) and one cycle (1W) of wet incubation. The cycle 1D sample is represented by one broad peak at ~2.18 min and smaller broad shoulders at ~2.3 and ~2.4 min probably due to high dispersity of the sample. The cycle 1W sample shows an additional shoulder at ~2.10 min that is due to monomeric citric acid confirmed by co-injection. It is unusual in SEC for the monomer to elute faster than the polymer. In this case, the phenomenon is probably due to the formation of extensive hydration shell around the citric acid molecule or the formation of non-covalent complexes. Glycerol monomer was not detected as it is invisible to the UV at 220 nm. Figure 1B shows the chromatographs of the wet-dry cycled samples collected at the end of the wet cycles [series W]. While the chromatogram 1D (Figure 1A) showed no peak due to the citric acid monomer, about 50% of all product in 1W samples is the citric acid monomer. Citric acid monomer does not appear in samples 2, 4, and 8W. In samples 2 and 4W the major peak is shifted compared to the cycle 1D sample from ~2.18 min to ~2.14 min possibly due to some polymer degradation. In the sample 8W the major peak remains at 2.18 min. Figure 1C compares between the samples belonging to DRY series. Qualitatively, all chromatograms look the same with major peak at ~2.18 min with shoulders at ~2.3 and ~2.4 min. The intensity of the peaks gradually diminishes with every successive sample due to partial precipitation. Only water-soluble fraction was analyzed. Finally, Figure 1D compares between the samples of the D series. Samples 1,2, and 4D appear roughly similar. Sample 8D here was analyzed after the normal workup of stirring the sample in water at room temperature for 12 hrs, therefore a very small fraction is visible in the chromatogram. The sample was fully dissolved after 48 h incubation at 75 °C (sample 8W).

### 3.3. Mass Spectrometry

**Figure 2:**
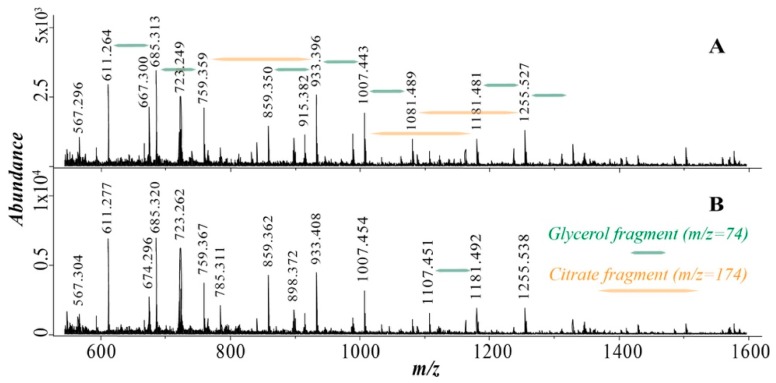
MALDI mass spectra of (**A**) the water-soluble fraction of the continuously dried sample collected after 8 periods (8DRY) and (**B**) cycled sample collected at the end of the experiment (8W). All labeled species correspond to (M + 23)^+^ ions (sodium adducts).

Figure 2 shows the MALDI mass spectra of (A) water-soluble fraction of the polymeric sample collected after a period of continuous drying for 384 h (cycle 8DRY, Scheme 2) and (B) polymeric sample that has undergone the most extended period of wet-dry cycling (cycle 8W, Scheme 2). Multiple masses consistent with glycerol-citric acid units detected as sodium adducts have been detected. A list of detected oligomeric species long with their abundances normalized to the abundance of the oligomer containing two glycerol and two citrate units (G_2_C_2_) is provided in the Table 1. The continuously dried sample (8D) contains significantly higher abundances of higher molecular weight oligomers as well as masses consistent with macrocyclic species than the cycled sample (8W). Neither of the mass spectra contains a signal corresponding to dicitrate implying that the hydroxyl group of the citric acid does not participate in the esterification reactions. The products in both spectra correspond to species enriched in glycerol consistent with the excess of glycerol in the starting material. Similar spectra have been obtained utilizing electron spray ionization (Figure S2).

### 3.4. Nuclear Magnetic Resonance (NMR)

**Figure 3:**
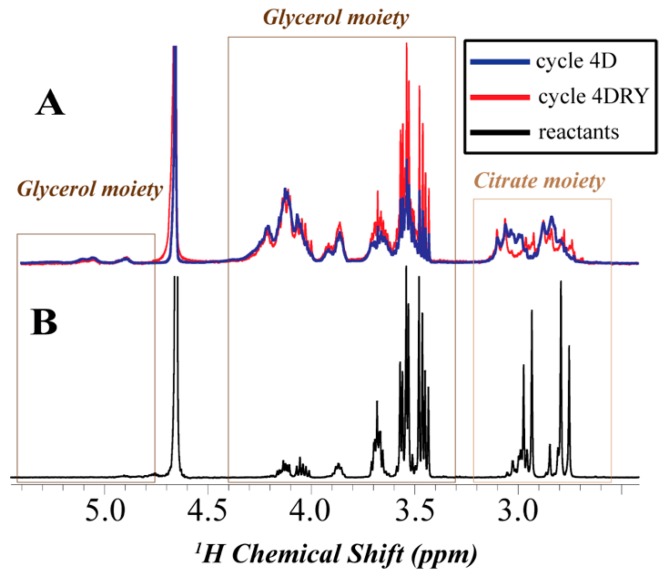
Representative ^1^ H NMR spectra of the glycerol-citric acid polymerization products in D2O along with general peak assignments. (**A**) The overlaid spectra of the soluble fraction of the 4DRY sample [red] and the 4D sample [blue]; (**B**) The spectrum of the unreacted sample.

Figure 3 shows representative ^1^H NMR spectra in D_2_O of the polymeric samples exposed to continuous drying and dry-wet cycling [Figure 3A] as well as unreacted citric acid and glycerol [Figure 3B] The quadruplet characterizes the spectrum of unreacted glycerol and citric acid [Figure 3B] at ~2.8 ppm corresponding to methylene hydrogens of citric acid, multiplets at ~3.4, 3.5, and 3.6 ppm representing methylene and methanetriyl hydrogens of glycerol. Small broad peaks associated with polymeric products are also represented in the spectrum. The unreacted sample as well as the rest of the sample has undergone a workup procedure that included a period of freeze-drying that is potentially conducive to the polyester formation. The spectrum of the sample that have undergone long wet incubation [8WET] closely resembles the spectrum of the unreacted solution [Figure S3] suggesting that polymerization did not occur during the wet incubation. The spectra of the polyesters [Figure 3A] is described by broadened, downfield-shifted signals corresponding to citric acid unit methylene hydrogens, broadening of the glycerol unit hydrogens, and the appearance of broad signals at ~4 and 5 ppm consistent with methylene and methanetriyl hydrogens at the RCOO-CH position. The protons of the carboxylic acid and alcohol groups were not detected. The signal assignments performed using prediction software and standards are described elsewhere [42]. The relative integrations of these signals suggest that only a small fraction of the hydroxyls [>6 %] at the 2 position of glycerol are esterified possibly due to steric hindrance. The line broadening can be explained either by the presence of multiple oligomeric isomers with overlapping signals or by slowed molecular tumbling of polymeric species.

Table 2 shows the relative integrations, normalized to the number of non-exchangeable protons, of the sum of the signals corresponding to citric acid and glycerol moieties. In all the cycled samples, the initial stoichiometry of two glycerols to one citrate is roughly preserved. Interestingly, the relative integrations suggest that the soluble fraction of the continuously dried samples appear to be enriched in glycerol suggesting that the insoluble fraction is enriched in citric acid. It has been shown [51] that glycerol-citric acid polymer prepared at 1:1 ratio is more prone to crosslinking and formes an insoluble product more easily than systems with an excess of glycerol. Slightly higher glycerol excess seen in cycles 4D/W and 8D/W possibly due to some precipitation. Most considerable excess of glycerol seen in the water-soluble fraction of 4DRY [Table 2, Figure 3]. These observations suggest that during continuous drying polymer containing the similar stoichiometry of citric acid and glycerol are formed first; the excess of glycerol incorporates later. The result suggest that wet-dry cycling does not only control the molecular weight of the product but the polymer make up as well.

## 4. Discussion

Planetary surfaces, lacking the enzymatic control of the biosphere or the synthetic chemist’s skill in the lab, generically produce combinatorially complex, heterogeneous mixtures of compounds. For example, in prebiotically plausible settings, self-polymerization of small molecules such as formaldehyde [58] or HCN [20] yields an intractable mixture and has been referred to as “tar” or “asphalt” [21]. In the chemical evolution studies, the production of the tarry material is often seen as an unwanted process, or even paradox implies that the workability of elucidating life’s origins might be an insuperable goal. The system of branched polyesters described above is a somewhat simplified representation of tar forming chemical system. However, gelation of branched polymers could be one of the mechanisms responsible for the tar generation. In the case of the branched polyesters, the onset of gelation or tar formation is delayed by wet-dry cycles. The results presented here indicate that wet-dry cycles associated with day-night, tidal, or seasonal environmental changes promote the formation of soluble lower molecular weight HBPE whereas continuous drying is conducive to the formation of insoluble polymers. Periods of intermittent wetting trigger the partial hydrolysis controlling the chain growth of the HBPEs and therefore retarding the gelation process.

Soluble HBPEs oligomers have been shown to possess enzyme-like catalytic activities. Therefore, a process of their synthesis and maintenance in the water solution can significantly affect abiotic systems. Furthermore, the NMR studies suggest that that during the continuous drying HBPE enriched in citric acid, more prone to crosslinking, are produced preferentially. We have previously demonstrated that HBPEs can be synthesized by subjecting multifunctional organic acids and alcohol mixtures to mild heating under dry conditions [42]. This method, however, produces a multitude of polymeric products varied in size and shape. One possible way of selecting for the desired HBP shape and function is to investigate further the concept of far-from-equilibrium polymerization by subjecting the polyesterification system to intermittent drying and wetting. Period of heating the open vessel stimulates esterification. Even though periodic sample rehydration promotes hydrolysis, successive iterations of wet-dry cycles result in polymer yields, and molecular weight distributions above that observed after heating alone. Products less prone to hydrolysis would tend to persist in the system at the expense of the rest. The hydrolysis patterns of HBPEs differs from linear polymer ones. The globular nature of HBPEs prevents or delays the water intrusion into the core, slowing down the hydrolysis process and resulting in macromolecular surface erosion rather than breakdown. When wet-dry cycling applied to HPBE condensation, the first drying phase will result in a mixture of linear, branched and mixed polymers. During the hydrated periods, linear portions of the polymer would be more susceptible to hydrolysis than their branched counterparts. Therefore, it is reasonable to assume that after some cycles the makeup of the polymer would consist predominantly of branched structures.

Recently, prebiotic systems chemistry [59,60,61] has begun to explore the processes of selection and organization in these heterogeneous, or “messy” systems in an attempt to understand the natural transition between the messy prebiotic chemistry and the well-controlled biochemistry, in other words, the transition between chemistry and biology. The mechanisms for the organization in messy chemistry that have been explored thus far include the emergence of autocatalytic sets [62,63,64], molecular imprinting [65] as a mechanism for heredity in chemical systems, as well as specific selective processes [66,67,68]. Herein the environmentally driven selective formation of different HBPE structures is explored. As the NMR analysis suggests, when the wet-dry cycling is employed products with a higher degree of branching are formed. Furthermore, mass spec results suggest that continuously dried samples produce higher quantities of macrocyclic species. The macrocyclic species might be more prone to hydrolysis than the branched oligomers and therefore do not persist throughout successive wetting cycles. The overall results indicate that depending on the reaction conditions, the product can be selectively enriched in species of three possible architectures, crosslinked, branched, and macrocyclic.

Analytical challenges have discouraged many other researchers from studying the inherent complexity of the prebiotic systems chemistry [61]. Experimental studies of the complex prebiotic systems are challenging to identify all the components unequivocally. The system described here is somewhat simplified; it starts with only two reactants being connected through one chemical bond with minimal side reactions, such as water eliminations. Nevertheless, due to the production of a multitude of branched architectures, the spectroscopic and chromatographic methods produce broad unresolved signals preventing the clear identification of every component in the system. Comparative analysis between different experimental conditions in addition to the bulk properties of the products permits to derive some structural information. While it is not necessarily feasible to produce precise, complex biological structures abiotically, a functional measurement to establish whether the messy products have a particular bulk property, i.e., solubility, color, swelling in a solvent, or capable of a particular function, i.e., catalytic ability, capability to absorb specific molecules, is a reasonable approach to hypothesis of the origin of life.

## 5. Conclusions

The results described above describe the polymerization ion citric acid-glycerol polyester formation to form a branched polyester. The polymerization was conducted under continuous drying and subjected to wet-drying cycles. The continuous drying yielded an insoluble glass-like swellable in water product after 48 h. In contrast, the system subjected to wet-dry cycling yielded a water-soluble product after 768-h experiment. The NMR studies indicated that the insoluble product was enriched in citric acid, a stoichiometry that favors crosslinking. While the analysis has proven challenging as expected in abiotic mixtures with significant heterogeneous content, the two experimental setups have produced polymeric products with different properties. In branched systems, the intermittent wetting has provided means to control the chain growth and delayed the gel transition. The described system is a model for further exploration of the formation of functional polymers under prebiotically plausible conditions.

## Figures and Tables

**Table 1:** Assignments and relative abundances of the MALDI mass spec signals.

Oligomer	m/z (+Na)	Normalized Abundance in 8D Sample ^2^	Normalized Abundance in 8W Sample ^2^
G_2_C_2_^1^	537.2	1	1
G_3_C_2_	611.3	2.50	1.87
G_4_C_2_ *	667.3	0.71	0.27
G_4_C_2_	685.3	2.93	1.88
G_5_C_2_	759.4	1.78	0.94
G_4_C_3_	859.4	1.22	1.14
G_5_C_3_ *	915.4	0.96	0.41
G_5_C_3_	933.4	2.18	1.21
G_6_C_3_ *	989.4	0.98	0.32
G_6_C_3_	1007.4	1.63	0.85
G_7_C_3_	1081.5	0.82	0.30
G_5_C_4_	1107.5	0.47	0.37
G_6_C_4_	1181.5	0.82	0.31
G_7_C_4_ *	1237.5	0.54	0.14
G_7_C_4_	1255.6	1.01	0.52
G_8_C_4_	1329.6	0.67	0.29

^1^ G_2_C2 refers to oligomer containing two glycerol (G) and two citrate (C) units. ^2^ The abundances are normalized to the absolute abundance of G_2_C_2_. * Mass Consistent with macrocyclic oligomers.

**Table 2:** Relative stoichiometries of non-exchangeable protons in citrate and glycerol moieties in the different polymeric products based on ^1^H NMR signal integrations. In the DRY series, only visibly water-soluble fraction is analyzed.

Series/Cycles	DRY	D	W	Initial	WET
0				4H:9.93H (1**C**:2.0**G**)	
1	4H ^1^:9.89H (1**C**:2.0**G**)	4H:9.89H (1**C**:2.0**G**)	4H:9.81H (1**C**:2.0**G**)		
2	4H:10.14H (1**C**:2.0**G**)	4H:9.94H (1**C**:2.0**G**)	4H:9.92H (1**C**:2.0**G**)		
4	4H:14.03H (1**C**:2.8**G**)	4H:10.20H (1**C**:2.0**G**)	4H:10.28H (1**C**:2.1**G**)		
8	4H:11.99H (1**C**:2.4**G**)		4H:10.37H (1**C**:2.1**G**)		4H:9.74H (1**C**:1.9**G**)

^1^ The 4 citrate non-exchangeable protons were set as constant; the glycerol signal integrals were measures relative to citrate signal integrals. The derives relative quantities of citric acid units (C) and glycerol units (G) are given in parentheses.

## Supplementary Materials

The following are available online at https://www.mdpi.com/2075-1729/9/3/56/s1, Figure S1: Normal phase SEC analysis of the citric acid glycerol polymerization products, Figure S2: ESI mass spectra Figure S3: 1H NMR spectra of citric acid glycerol polymerization controls.
