# Supplementary material for: Wet-Dry Cycling Delays the Gelation of Hyperbranched Polyesters: Implications to the Origin of Life
Source: Life (Basel). 2019 Jul 1;9(3):56. doi: 10.3390/life9030056 (PMC6789768; doi:10.3390/life9030056)
Supplement: Supplementary file 1 [file life-09-00056-s001.pdf]

Wet-dry Cycling Delays the Gelation of Hyperbranched Polyesters: Implications to the Origin of Life

Irena Mamajanov [1,*]

[1]Earth-Life Science Institute, Tokyo Institute of Technology, Meguro, Tokyo 152-8550, Japan; irena.mamajanov@elsi.jp

*Correspondence: irena.mamajanov@elsi.jp

Supplementary Material

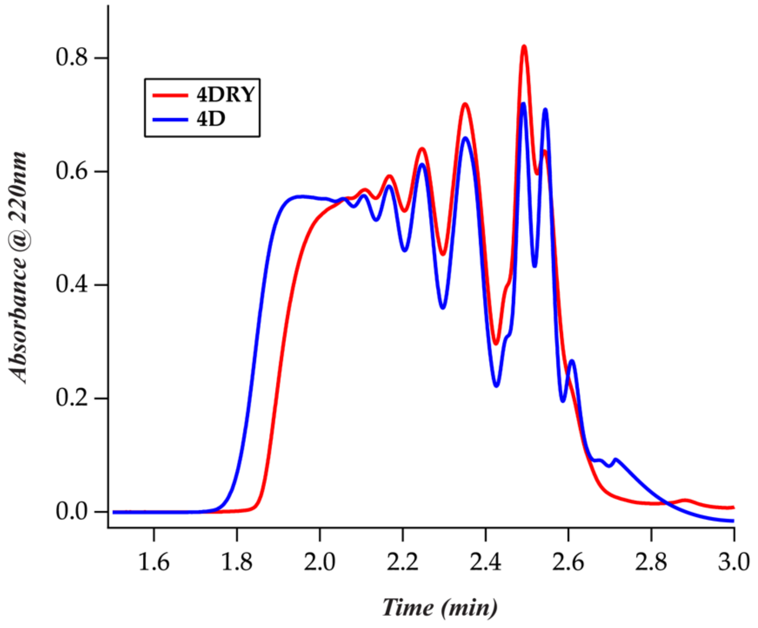

Figure S1. SEC analysis of the citric acid glycerol polymerization products. Sample collected after 4 periods of continuous drying (4DRY) cycle is in red, sample collected after four cycling period (4D) is in blue.

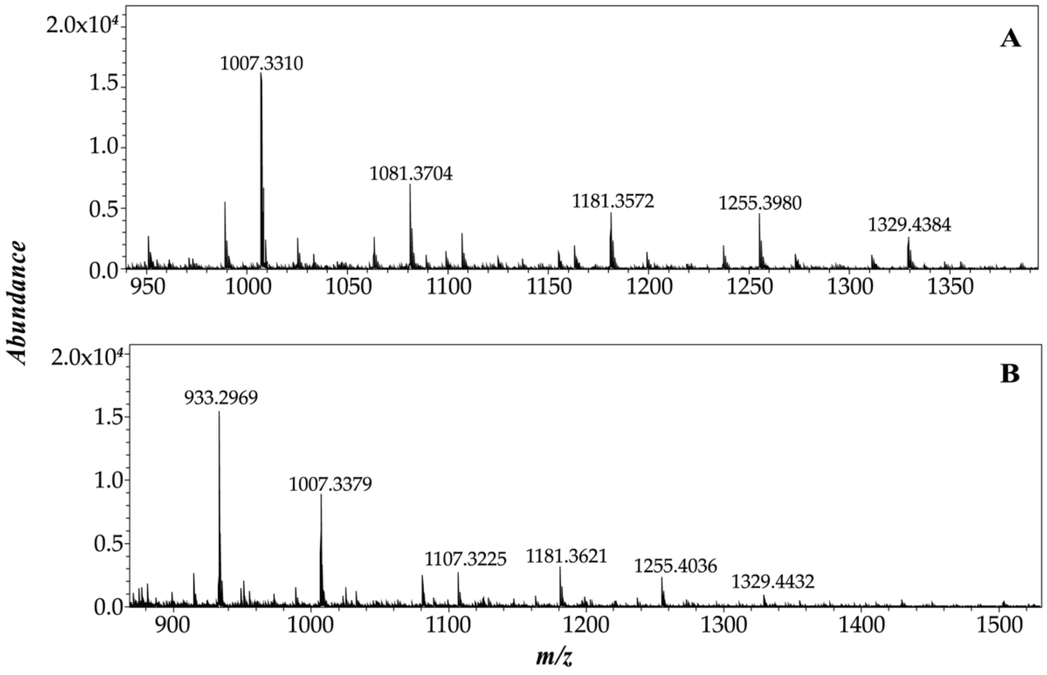

Figure S2. ESI mass spectra of a) the water-soluble fraction of the continuously dried sample collected after 8 periods (8DRY) and (b) cycled sample collected at the end of the experiment (8W). All labeled species correspond to $(M+23)^+$ ions (sodium adducts).

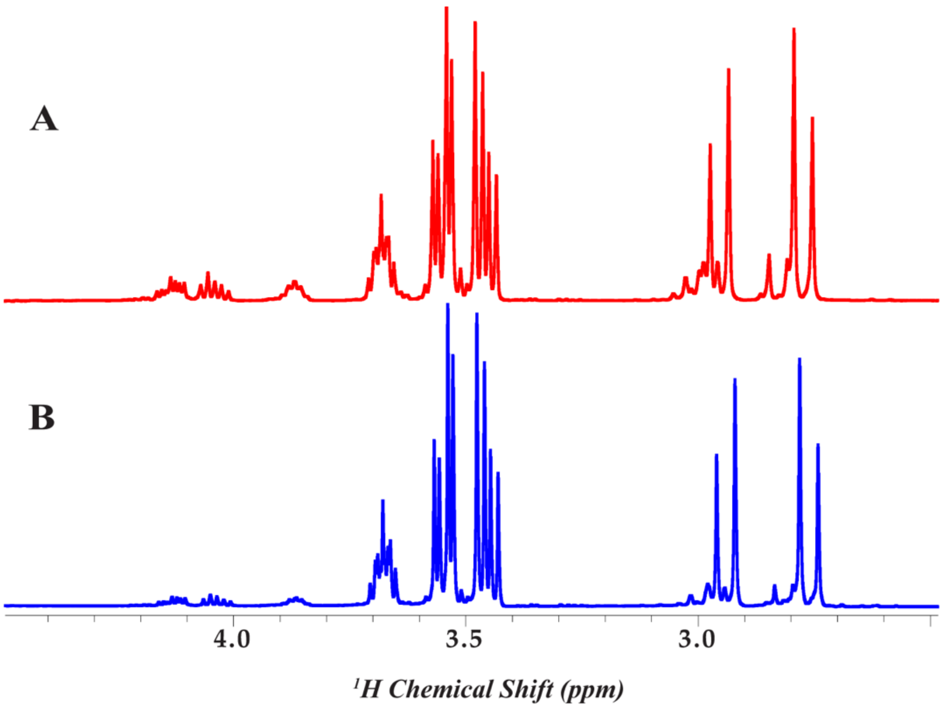

Figure S3. ^{1}H NMR spectra of the glycerol-citric acid polymerization contols. (a) Unreacted sample (b) The sample after the wet incubation (8WET)
